# Senescence-Associated Secretory Phenotype as a Hinge Between Cardiovascular Diseases and Cancer

**DOI:** 10.3389/fcvm.2021.763930

**Published:** 2021-10-20

**Authors:** Priyanka Banerjee, Sivareddy Kotla, Loka Reddy Velatooru, Rei J. Abe, Elizabeth A. Davis, John P. Cooke, Keri Schadler, Anita Deswal, Joerg Herrmann, Steven H. Lin, Jun-ichi Abe, Nhat-Tu Le

**Affiliations:** ^1^Center for Cardiovascular Sciences, Houston Methodist Research Institute, Houston, TX, United States; ^2^Department of Cardiology, The University of Texas MD Anderson Cancer Center, Houston, TX, United States; ^3^Department of Pediatrics Research, The University of Texas MD Anderson Cancer Center, Houston, TX, United States

**Keywords:** SASP, senescence associated secretory phenotype, cancer, cardiovascular disease, replicative senescence (RS), stress-induced premature senescence (SIPS)

## Abstract

Overlapping risks for cancer and cardiovascular diseases (CVD), the two leading causes of mortality worldwide, suggest a shared biology between these diseases. The role of senescence in the development of cancer and CVD has been established. However, its role as the intersection between these diseases remains unclear. Senescence was originally characterized by an irreversible cell cycle arrest after a high number of divisions, namely replicative senescence (RS). However, it is becoming clear that senescence can also be instigated by cellular stress, so-called stress-induced premature senescence (SIPS). Telomere shortening is a hallmark of RS. The contribution of telomere DNA damage and subsequent DNA damage response/repair to SIPS has also been suggested. Although cellular senescence can mediate cell cycle arrest, senescent cells can also remain metabolically active and secrete cytokines, chemokines, growth factors, and reactive oxygen species (ROS), so-called senescence-associated secretory phenotype (SASP). The involvement of SASP in both cancer and CVD has been established. In patients with cancer or CVD, SASP is induced by various stressors including cancer treatments, pro-inflammatory cytokines, and ROS. Therefore, SASP can be the intersection between cancer and CVD. Importantly, the conventional concept of senescence as the mediator of cell cycle arrest has been challenged, as it was recently reported that chemotherapy-induced senescence can reprogram senescent cancer cells to acquire “stemness” (SAS: senescence-associated stemness). SAS allows senescent cancer cells to escape cell cycle arrest with strongly enhanced clonogenic growth capacity. SAS supports senescent cells to promote both cancer and CVD, particularly in highly stressful conditions such as cancer treatments, myocardial infarction, and heart failure. As therapeutic advances have increased overlapping risk factors for cancer and CVD, to further understand their interaction may provide better prevention, earlier detection, and safer treatment. Thus, it is critical to study the mechanisms by which these senescence pathways (SAS/SASP) are induced and regulated in both cancer and CVD.

## Introduction

**Figure 1:**
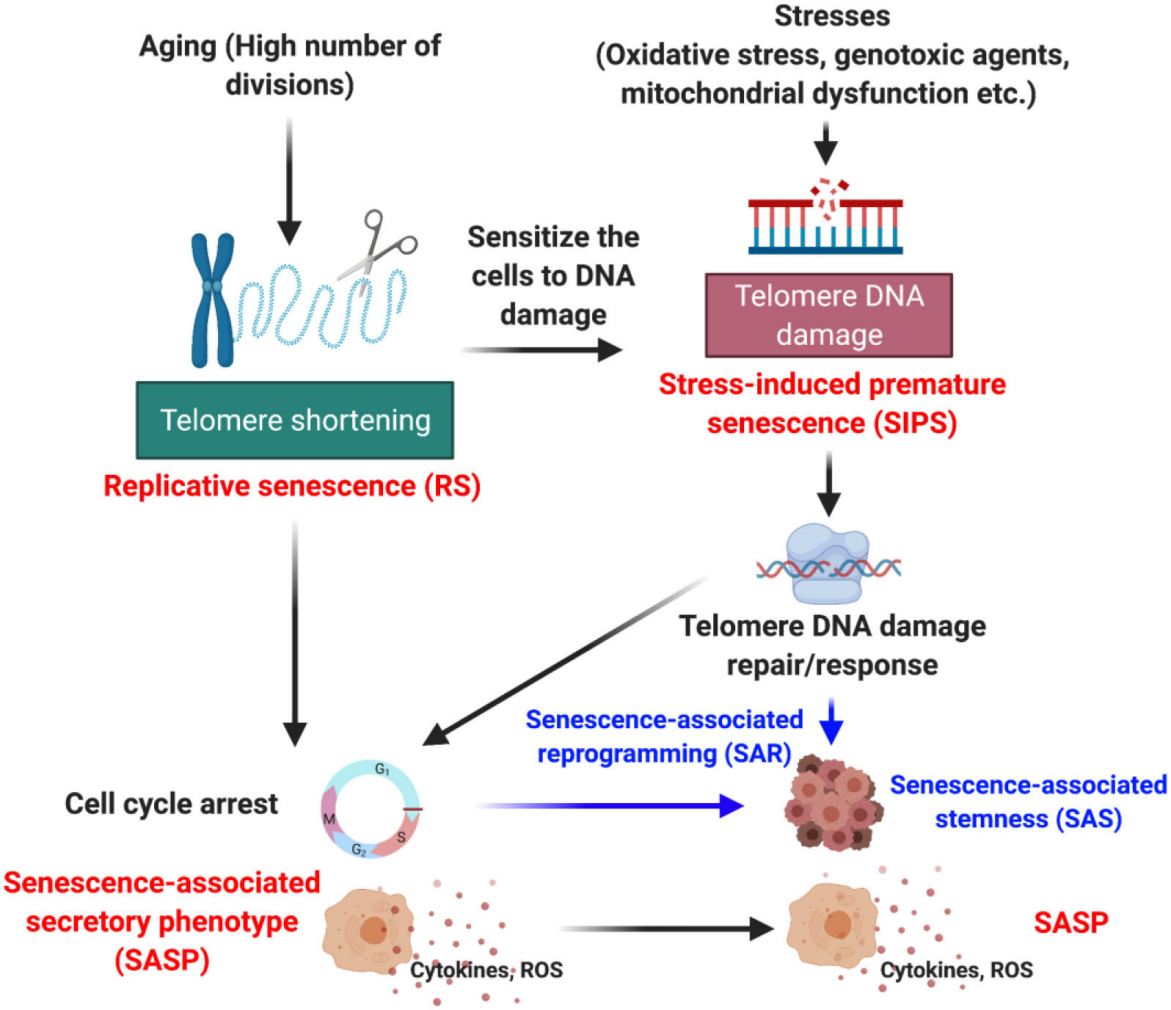
Various forms of senescence.

The health and physiological state of humans or any animal is governed by tissue homeostasis which is significantly controlled by physiological and environmental signals (1, 2). In response to potential damage signals, cellular machinery activates the damage response system to reverse damage to the cells through various mechanisms, as have been reviewed extensively elsewhere (3–5). However, when the damage is irreparable, the cells often undergo a programmed cell death, or apoptosis, in combination with tissue necrosis (5). Distinct from these two extreme phenomena is another cell fate called “senescence” (6, 7). The concept of cellular senescence (from the Latin word “senex” meaning “old”) was first introduced by Hayflick and Moorhead in 1961 when they observed that in cell culture, human diploid fibroblasts were irreversibly arrested after serial passaging (8). This limited replicative/proliferative capacity was named replicative senescence (RS) (8, 9). In addition to RS, cellular senescence can be induced by both extra- and intra-cellular stimuli including genotoxic agents, stress, mitochondrial dysfunction, nutrient deficit, radiation, and oncogene activation, so-called stress-induced premature senescence (SIPS) (Figure 1). In this review, we will focus on how senescence, especially SIPS, contribute to the progression of cancer and cardiovascular diseases (CVD), which may be the key to understanding the interconnection between them.

## Telomeric DNA Damage, But Not Telomere Shortening, Induces Sips

Following cell divisions, telomere length is shortened to a critical level at which cells can no longer replicate and enter RS (9–13). Therefore, telomere shortening has a key role in RS. Alternatively, SIPS is different from RS in terms of molecular mechanisms and time frame. SIPS is induced by oxidative stress or DNA damaging agents in a relatively short period of time (usually 3–10 days) with or without significant telomere shortening (14). Both genomic and telomeric DNA damages can induce SIPS (15). However, most genomic DNA damages can be repaired by the DNA damage response (DDR) mechanisms within 24 h after stress (16), while telomeric DNA damages persist for months (17). Therefore, telomeric DNA damage-induced SIPS may explain the late effects triggered by various stressors including cancer treatments, as we will describe in the next sections. Importantly, telomeric DNA damages are occurred despite the shortening of telomere length and the expression of telomerase enzyme (17, 18). The dispensable role of telomere shortening in the development of senescence was also confirmed by the study showing that in human cancer cells, the very long telomeres were found to be more sensitive to ionizing radiation (IR) (19). Parrinello et al. reported that 20% oxygen density induces SIPS in mouse embryonic fibroblast without telomere shortening (14). Magalhães et al. reported that Ultraviolet B or hydrogen peroxide (H_2_O_2_) induces senescence markers, p21(WAF-1) and p16(INK4a), and increases senescence associated β-galactosidase (SA-β-gal) staining without provoking telomere shortening in telomerase immortalized human foreskin fibroblast, hTERT-BJ1 (20). An analysis of the telomere in the small airway epithelial cells from the lungs of patients suffering from Chronic Obstructive Pulmonary Disease showed that p16(INK4a) was highly expressed in those cells while the telomere length was not significantly shorter (21). Overall, these data suggest that telomere shortening is dispensable for SIPS, and that stress-induced telomeric DNA damages and the subsequent DDR, but not telomere shortening, is important for SIPS (20).

## Senescence-Associated Secretory Phenotype (SASP) Can be Induced by Both RS and Sips

Senescent cells produce and release a variety of factors, including inflammatory cytokines (such as interleukin (IL)-1,−1b,−6,−7,−13, and−15), chemokines (IL-8, grow regulated alpha protein 1 (GRO)-a, -b, and -g, monocyte chemoattractant protein (MCP)-2 and−4, macrophage inflammatory protein (MIP)-1a and−3a, human beta C-C chemokine-4 (HCC-4), eotaxin, eotaxin-3, thymus-expressed chemokine [TECK, also known as C-C motif chemokine ligand-25 (CCL-25)], C-X-C motif chemokine-5 (CXCL-5 or ENA78), CCL-1 (or I-309), CXCL-11 (or I-TAC), growth and angiogenic factors [such as amphiregulin, angiogenin, epiregulin, heregulin, epidermal growth factor (EGF), basic fibroblast growth factor (bFGF), hepatocyte growth factor (HGF), insulin-like growth factor binding proteins (IGFBP)-2,−3,−4,−6, and−7, keratinocyte growth factor (KGF), nerve growth factor (NGF), placenta growth factor (PIGF), stem cell factor (SCF), stroma cell-derived factor-1 (SDF-1), vascular endothelial growth factor (VEGF)], matrix metalloproteinases (MMP)-1,−3,−10,−12,−13, and−14, metallopeptidase inhibitor (TIMP)-1 and−2, plasminogen activator inhibitor (PAI)-1 and−2, tissue plasminogen activator (tPA), urokinase-type plasminogen activator (uPA); and cathepsin B, receptors/ligands [EGF receptor, Fas ligand, intercellular adhesion molecule (ICAM)-1 and−3, osteoprotegerin (OPG), uPA receptor, soluble gp130 protein (SGP130), soluble tumor necrosis factor receptors (sTNFRs including sTNFR-I and sTNFR-II, and decoy receptor 1 (DCR-1, also known as TRAIL-R3), non-protein molecules (including nitric oxide (NO), prostaglandin E2 (PGE2); and reactive oxygen species (ROS)], and insoluble factors (collagens, fibronectin, and laminin), all of which constitute SASP (22, 23) (Figure 1). Senescent cells undergoing SASP have high metabolic activity (24–27). Although the consequence of SASP can be multifarious, the induction of SASP does not depend on the type of triggers such as ROS, DNA damage, oncologic signaling, or cell types (24). First report of SASP was described in human fibroblasts undergoing RS, which showed a strong inflammatory response by using microarray analysis (22). SASP components including IL-6 and−12, MIP-2, and interferon-gamma (IFN-g) were similar between RS and SIPS fibroblasts, suggesting that SASP can be induced by both RS and SIPS (Figure 1) (22, 24). By inducing SASP, senescent cells communicate with immune cells playing a role in their own death, through recruitment of T cells, macrophages, and natural killer cells, which function collectively to clear the senescent cells. To maintain tissue homeostasis, the removal of senescent cells in a timely manner is crucial. With aging, the immune response declines, a phenomenon known as “immunosenescence” (28). As a result, the clearance of senescent cells is impaired.

SASP cells also can recruit myeloid derived suppressor cells (MDSCs), a heterogeneous and immature population of myeloid cells that can suppress immune responses, to prostate and liver tumors and accelerate tumorigenesis. For example, CCL-2, an important SASP factor secreted by senescent cells, attracts MDSCs to the tumor site. In the presence of tumor derived factors, MDSCs fail to differentiate and inhibit the function of other immune cells, such as T cells, dendritic cells, macrophages, and natural killer cells, and thereby creating an immune tolerant environment (28–31).

In addition to communicating with immune cells, by secreting extracellular vesicles (EVs), senescent cells undergoing SASP also communicate with surrounding cells to promote senescence in these neighboring cells (32–36). EVs or small heterogeneous vesicles are secreted from stressed or activated cells as result of cytoskeletal reorganization (37). EVs are characterized into exosomes (nanometers, <120 nm), micro vesicles (or microparticles, 100–500 nm), and apoptotic bodies released upon fragmentation of apoptotic cells (larger size, 500–5,000 nm) (38). EVs contain proteins, lipids, and nucleic acids (mRNA, DNA, and non-coding RNAs such as microRNAs and long non-coding RNAs). EVs are detected in biological fluids, enriched in specific proteins and lipids, and are produced by all cell types. For instance, through cell-cell interactions and secretion of soluble molecules, mesenchymal stem cells (MSCs) exert their functions on surrounding cells. These functions include anti-inflammation, anti-fibrosis, anti-apoptosis, pro-proliferation, and pro-angiogenesis. Among factors that are secreted by MSCs, PGE2, transforming growth factor-β (TGF-β), IL-6, IL-1 receptor antagonist (IL-1RA), tumor necrosis factor (TNF)-inducible gene 6 protein (TSG6), NO produced by inducible NO synthase (iNOS), or kynurenine produced by indoleamine 2. 3-dioxygenase (IDO) are part of the anti-inflammatory secretome. Other molecules, including HGF, FGF, and VEGF are important components of MSC paracrine activity, which are primarily generated within EVs that have a key role in cell-cell communication (39). With aging, the production of EVs is increased, partly *via* mechanisms dependent on p53 and its downstream target gene tumor suppression-activated pathway 6 (TSAP6). In endothelial cells, compared to the lower passage (passage 4) and non-senescent cells, the higher passage (passage 21) and activated senescent cells produce an increased number of functional small EVs, which may have a role in vascular physiology and disease (37, 40). Microparticles secreted by senescent endothelial cells increase ROS production and enhance the senescence of neighboring endothelial cells. In addition to that, microparticles also increase the expression of cellular senescent markers p21(WAF-1) and p16(INK4a) in endothelial cells (37, 41).

The induction of SASP is a highly heterogeneous, multi-step, and dynamic process, during which the properties of senescent cells continuously evolve and diversify in a context dependent manner (7). The SASP component is associated with the duration of senescence, the type of senescence stimuli, as well as the cellular origin (42). Long-term persistence of SASP and senescent cells has been shown to promote the development of CVD, cancer, aging-related disease, and aging itself (43).

## Senescence-Associated Stemness (SAS), A Unique Phenotype of SASP, is Induced by Sips

Senescence is characterized by cell cycle arrest, and therapy-induced senescence has long been the basis for cancer treatments to inhibit cancer cell growth (25). Recently, this conventional concept has been challenged (44–46). Milanovic et al. reported that senescent cancer cells induced by chemotherapy can be reprogrammed to acquire “stemness” (SAS: senescence-associated stemness), which allows them to escape senescence-mediated cell cycle arrest (Figure 1). Importantly, these senescent cells, which escape cell cycle arrest, gain an elevated tumor-initiating capacity possessing enhanced clonogenic growth potential compared to cells that have never undergone senescence (45). SASP is different from senescence-mediated cell cycle arrest (47, 48) and death (49, 50). SASP exerts a range of tumorigenic effects, including promoting angiogenesis, invasion, and metastasis (7) and is now considered one of the key mechanisms in the development of chemoresistance (7, 49–51). For instance, previous study has reported that through producing WNT16B, chemotherapy-triggered damage of stroma fibroblast promotes therapy resistance (52). Although the exact molecular mechanisms by which SASP induces cancer therapy resistance remain elusive, it is possible that SASP triggers the formation of cancer stem cells and thereby eluding drug treatment and reproducing tumor (53). Another possibility is that SASP causes cancer treatment resistance through inducing MDSC-driven immune-suppression (28). Therefore, the concepts of SASP and SAS largely overlap (Figure 1), but they describe different biological phenomena. After cancer therapy, subsets of senescent cells produce numerous inflammatory cytokines (54), growth factors (23), ROS (55–57), and promote cell growth (24, 58), eventually leading to a more aggressive proliferative phenotype (45, 49, 50) through SAS (59, 60). Although SASP can be induced by both RS and SIPS, it remains unclear whether RS can provoke SAS.

## Stress Reprograms Cells to SASP, Locking In and Leading to the Long-Term Effects of SASP

Pro-inflammatory stimuli increase inflammation, but these effects are temporary. The uniqueness and probably most important feature of SASP is its long-term effects to the cells. In fibroblast, Coppé et al. have suggested that a large proportion of SASP produced by senescent fibroblasts is irreversible once established. In these cells, the inactivation of p53 can reverse the growth arrest and resume the cell proliferation, despite the low level of p16(INK4a). These findings suggest that SASP might be permanently locked in an irreversible stage by unknown mechanisms that uncoupling senescence-associated cell cycle arrest from the SASP (24).

Oncogene-induced senescence (OIS), characterized by marked epigenetic changes, can promote tumor progression (23). *Via* evaluating changes in histone modifications during the senescence of the OIS classic cell model, HRAS^G12V^ overexpression in IMR90 cells, or RAS (61), Leon et al. observed an increase of active histone H3K79 di- and tri-methylation (H3K79me2/3) marks at the IL1A locus. This increase corresponds to an increase of the H3K79 methyltransferase disruptor of telomeric silencing 1-like (DOT1L) expression. In OIS cells, DOT1L upregulation is required for H3K79me2/3 and *IL1A* expression. Knockdown of DOT1L during RAS-induced senescence decreases both DOT1L and H3K79me2/3 occupancy at the *IL1A* locus but not at other SASP loci, and decreases the expression of *IL1A* mRNA, the epxresison of IL1A at the cellular membrane, as well as the transcription and secretion of SASP downstream factors. Leon et al. also found that the decrease of SASP was not due to the rescue of senescence-associated cell cycle arrest. Although DOT1L can regulate DDR, and H3K79 methylation promotes 53BP1 binding to sites of DNA double-strand breaks, the authors found no marked changes in 53BP1 or γH2AX foci upon DOT1L knockdown. DOT1L knockdown in BRAF-induced senescent cells or pharmacological inhibition of DOT1L in RAS-induced senescent cells inhibited H3K79 methylation and SASP expression while maintaining the senescence-associated cell cycle arrest. These observations indicated that DOT1L regulates SASP through a DDR-independent mechanism, and that DOT1L expression is required for the SASP but is dispensable for other senescent cell phenotypes. Overall, this study suggested DOT1L as an epigenetic regulator of SASP, whose expression is uncoupled from the senescence-associated cell cycle arrest (62).

Various stresses can induce epigenetic changes and thereby leading to persistent changes in gene expression *via* inducing chromatin alteration (63). Because these stresses also induce SASP, as in the case of OIS presented above, epigenetic regulation can be one of the potential mechanisms that causes the irreversible stage of SASP (64, 65). Therefore, it is possible that epigenetic changes induced by SASP have some impact on the establishment of long-term effects of SASP. In mid-life flies, acetyl-CoA levels are increased with a corresponding increase in histone acetylation resulting in changes in their transcriptomes (66, 67). However, in mammals, the role of epigenetics in SASP remains unclear. For example, IR-triggered epigenetic changes have been extensively studied, but the results are contradictory (68). Therefore, SASP is potentially induced and maintained by various stresses rather than solely by epigenetic changes, which requires further investigation.

## SASP in Tumorigenesis: Foe or Ally?

**Figure 2:**
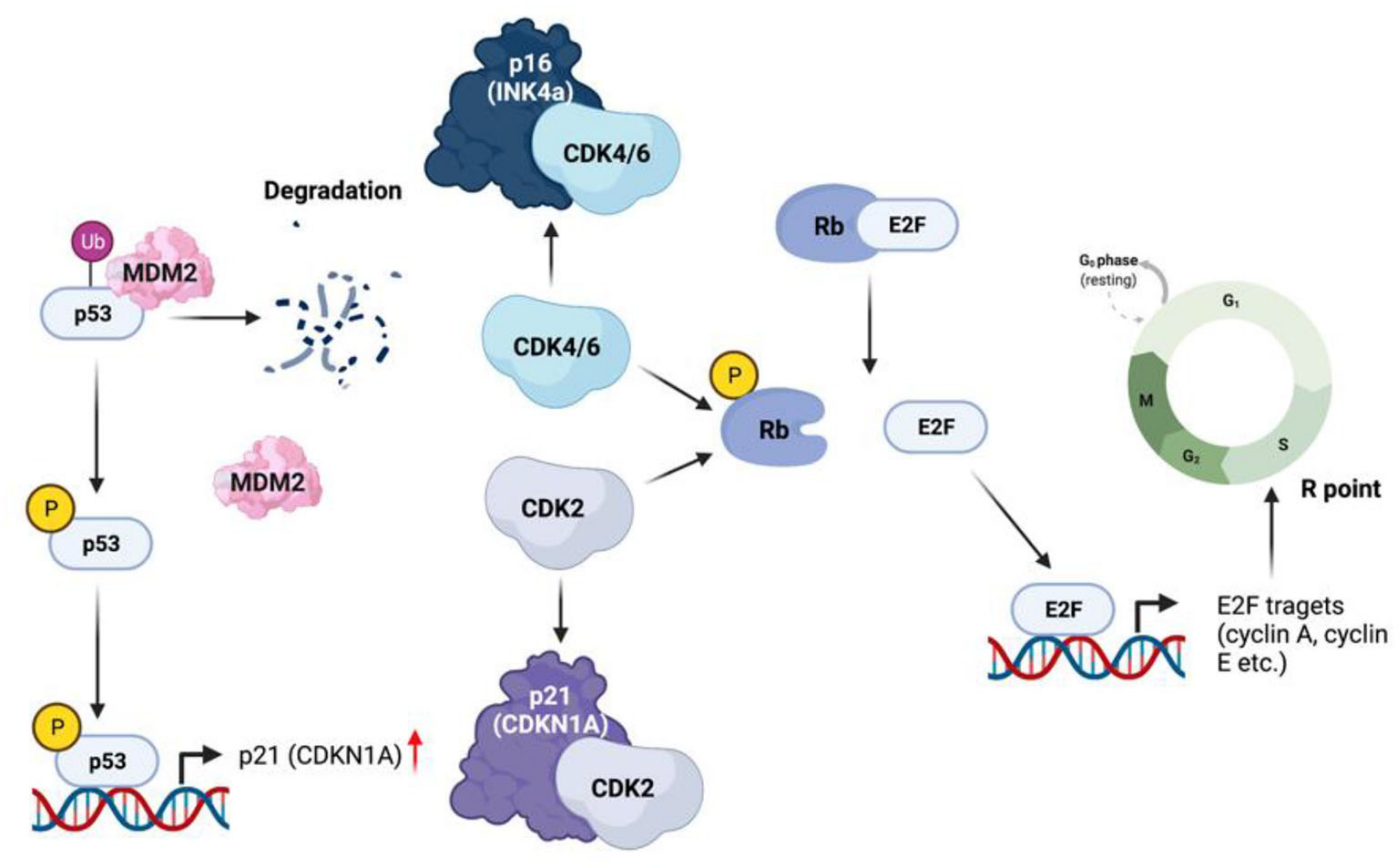
SASP in tumorigenesis.

The role of senescence in cancer is highly controversial. SASP can be induced in both cancer and normal cells by stress, oncogenes, or therapy (therapy induced senescence) (69, 70). However, recent studies of senescence have led investigators to consider this process as a double-edged sword for cancer (70). SASP is associated with changes in p16(INK4a), retinoblastoma (Rb), and p53. *Via* binding to the E2 factor (E2F), Rb blocks the transcription of several E2F targeted genes that are essential for DNA replication. Cyclin and cyclin dependent kinase CDK4/6 phosphorylate Rb, leading to the release of E2F, ultimately leading to cell cycle progression (71). As one of the most widely known markers of senescence, p16(INK4a) binds CDK4/6 and thereby keeping the cells growth arrested at G1 through inhibiting CDK4/6-triggered Rb phosphorylation (72). p53, another important transcription factors, also plays a vital role in the control of cellular senescence. In the inactive form, p53 is bound to Mdm2, an E3 ubiquitin ligase and destined to ubiquitination and proteasomal degradation (73, 74). In response to stress, p53 is phosphorylated, leading to its release from Mdm2 and activation. Activated p53 increases the transcription of several target genes, including p21^Cip1^ (CDKN1A), known as another potential marker of senescence. p21Cip1 binds and inhibits cyclin dependent kinase 2 (CDK2), resulting in the activation of Rb and cell cycle arrest (Figure 2). Thus, cellular senescence *via* the activation of p21Cip1, can lead to cell cycle arrest and inhibition of tumorigenesis (70).

SASP also attracts immune cells to the tumor site for immune clearance of cancer cells. In liver cancer, OIS hepatocytes secrete CCL-2 that attracts CCL-2+ myeloid cells to the tumor site. CCL2+ myeloid cells are de-differentiated to macrophages, engulf cancer cells, and clear them out. Therefore, SASP can also inhibit tumorigenesis by promoting phagocytosis of pre-malignant cells (75, 76).

Importantly, in cancer progression, the effects of SASP on surrounding cells is context dependent. SASP promotes cancer progression by mediating the de-differentiation and division of the neighboring metastatic cancer cells, or triggering an epithelial-to-mesenchymal transition, one hall mark of cancer. Ritschka *et al* reported that primary mouse keratinocytes treated with the conditioned media from oncogene-transfected senescent cells induce both senescence and stemness markers such as CD34, Lgr6, Prom1, CD44, Ngfr, and Nestin (77). Lluc Mosteiro et al. showed that c-Myc overexpression enhances both stemness and senescence in the neighboring cells by increasing IL-6 production (78). In addition, senescent cancer cells share similar secretory phenotypes with cancer associated fibroblasts, a functionally heterogeneous population of activated fibroblasts that constitutes a major component of tumor stroma (79).

Two sub populations of cancer associated fibroblasts, inflammatory cancer associated fibroblasts and myofibroblastic cancer associated fibroblasts (80), express smooth muscle actin and soluble factors that promote cancer cell motility and progression. The main tumor-promoting factor secreted by cancer associated fibroblasts is CXCL-12, which is also a component of SASP (81). CXCL-12 promotes cancer cell proliferation as well as the angiogenesis (82). Other common factors secreted by cancer associated fibroblasts and senescent cells include SDF-1, GRO-a and -b, IL-8, MCP-1 and−8, all of which contribute to the promotion of cancer progression (82–84). Senescent cancer cells also secret factors that can affect cancer associated fibroblasts in a paracrine manner and attract them to tumor sites. Lastly, *via* secreting matrix metalloproteinases, senescent cells can also restructure the extracellular matrix that can facilitate cancer growth and reduces contact inhibition (22, 85, 86). Therefore, it is possible that SASP cells can escape from cell cycle arrest and promote tumorigenesis, which is reported as SAS. Although this has become a widely studied topic, how SAS is initiated remains largely unclear.

SASP is also induced by various cancer treatments (87). Conventional cancer treatments include chemotherapy, radiotherapy, chemo-radiotherapy, and surgery. Chemotherapy is a type of standard cancer treatments. In the 1960's, the discovery of anthracyclines (daunorubicin, doxorubicin (DOX), epirubicin, idarubicin, mitoxantrone, and valrubicin) in Italy was a breakthrough in oncology. Despite dramatic changes in cancer treatments in subsequent decades, anthracyclines remain the cornerstone of contemporary chemotherapy for various cancers. In the late 1970's, bleomycin, vinblastine, and cisplatin were used in chemotherapy. From the early nineteenth century, cancer treatments include radical, super-radical and ultra-radical surgery. During 1891–1981, radical mastectomy was used in breast cancer treatments. However, in 1981, the use of radical surgery for cancer treatment was disapproved and was reduced as soon as the combination of systemic adjuvant therapy and local surgery was shown to produce similar results. Systemic adjuvant therapies include radiation and cytostatic drugs are required to treat cancer dissemination and metastasis. In the last 60 years, novel cancer treatments have been drastically developed, including targeted therapies using small molecule inhibitors and monoclonal antibodies (MAbs). Recently, two types of immunotherapies have significantly impacted oncology, including Checkpoint inhibitory MAbs and chimeric antigen-specific receptor (CAR)-transfected T-cells (CAR-T cells). Immunotherapy has been shown to produce durable responses in numerous tumor types. Antigen-specific immune responses can be markedly effective, even in late-stage disease. Additionally, two other types of biological therapies, antitumor vaccines, and oncolytic viruses, have been developed. They are physiological and well-tolerated (88).

Anthracyclines are well-known for both effectiveness and cardiotoxicity (89). The destruction of cardiomyocytes causes cardiac dysfunction. Therefore, cardiomyocytes have been considered the major target of anthracyclines (89). Widely used as a prototypical anticancer drug, DOX treatment causes cardiotoxicity and cardiac dysfunction through the mechanism involves the accumulation of ROS and reactive nitrogen species (RNS) in adult cardiac muscle cells. DOX also targets other cardiac cell types such as cardiac progenitor cells and cardiac fibroblasts. For instance, DOX detrimental effects on endogenous cardiac stem cell (CSC) pool was detected, which induces premature senescence. DOX increases ROS production and DNA damage in resident CSCs with the induction of senescence and apoptosis. DOX altered the myocardium of treated patients who exhibited a higher number of CSCs marked by DNA damage and senescence, particularly by the phosphorylated form of histone H2AX and p16INK4a. Beyond the toxicity on cardiomyocytes and other cardiac cell types, recent studies have suggested that other cell types, including endothelial cells, also play a role in the pathogenesis of anthracycline-induced cardiomyopathy (89, 90). There are two major types of cardiotoxicities caused by anthracyclines: acute and chronic forms. The acute cardiotoxicity occurs after a single dose or a single course of treatment, with symptoms developed within 14 days from the end of treatment. The chronic cardiotoxicity can be further divided into the early onset and the late onset. The early onset chronic cardiotoxicity occurs within a year after treatment, shown as a dilated-hypokinetic cardiomyopathy with progressive evolution toward heart failure. The late onset chronic cardiotoxicity occurs after years or decades from the end of treatment. While the acute cardiotoxicity usually is reversible, the two chronic forms are considered irreversible, with a poor prognosis and a limited to heart failure therapy. Although the specific mechanisms of anthracycline-induced cardiotoxicity remain to be fully elucidated, the involvement of ROS production, DNA damage, cellular senescence and cell death, changes in iron metabolism, and Ca2þ signaling has been suggested. During DNA replication, transcription, or recombination, topoisomerase (Top) 2β uncoils DNA filaments, triggers mitochondrial dysfunction, activates cell death pathways and ROS production. Top2β has been shown to play a critical role in anthracycline-mediated cardiotoxicity (89). Similarly, bleomycin, vinblastine, and cisplatin also induced severe side effects.

Advances in cancer treatments have significantly reduced morbidity and increased survival of cancer patients, but the side effects on non-cancer cells have significantly affected the quality of patient's life. Moreover, cancer cells acquire resistance to therapies and progress to become more aggressive (91–93). In addition to the cytotoxic or less direct cytostatic cancer therapies, one of attractive strategies for cancer treatments is to provoke cellular senescence, so-called therapy-induced senescence (94). Therapy-induced senescence creates a cytostatic effect and slows down the growth of cancer cells. However, therapy-induced senescence can also induce SASP, which may cause conflicting effects on tumorigenesis as previously noted. Therefore, a critical role of SASP-modulating therapies in cancer treatments has recently been recognized. Currently, there are two major strategies for SASP-modulating therapies. One strategy is to convert senescent cells from tumor-promoting to an anti-tumorigenic phenotype. For example, Toso et al. reported that, the Pten null mice develop Pten-loss-induced cellular senescence, which is characterized by an immunosuppressive SASP that promotes tumorigenesis (95). Alternatively, inhibition of Jak2/Stat3 signaling reprograms the SASP cytokine networks by restoring senescence surveillance and tumor clearance, which enhances chemotherapy efficacy. We listed other candidates of SASP-modulating therapies in Table 1. Another strategy is using senolytic compounds to selectively eliminate SASP cells through inducing apoptosis known as “senolysis” (118). This strategy was based on the observation that in contrast to non-senescent cells, SASP cells can activate survival pathways and therefore are highly resilient to apoptosis (119). These Senescent Cell Anti-Apoptotic Pathways (SCAPs) such as pathways that regulate Caspase-3 (120), Mcl-1, BLC-2, BCL-XL, and BCL-W etc. (121, 122), might be mediated by senescence-associated mitochondrial dysfunction (6). The upregulation of these anti-apoptotic pathways of SASP cells protect them from apoptotic stimuli including serum withdrawal (121), UV damage (122), oxidative stress (123), extrinsic apoptotic inducers (122, 124), and cytotoxic drugs such as staurosporine or thapsigargin (120, 125). Therefore, a majority of senolytic agents target these anti-apoptotic factors including BCL-2 family of proteins (BCL-2, BCL-XL, and BCL-W, Mcl-1), p53-p21Cip1 axis, hypoxia-inducible factor 1-alpha, heat shock protein 90, several receptor tyrosine kinases, and the PI3K/Akt/mTOR pathway (122, 126). Senolysis induced by glutamate metabolic enzyme GLS1 inhibitor selectively eliminates SASP cells from various organs and tissues and aged mice, ameliorates age-related tissue dysfunction and the symptoms of arteriosclerosis and obese diabetes (127). As SASP cells take weeks to reaccumulate, a “hit-and-run” approach can be used to administer these senolytic drugs. The first senolytic drugs discovered including Dasatinib, Quercetin, Fisetin, and Navitoclax transiently cause apoptosis to SASP cells. Using preclinical models, studies have shown that senolytic drugs can delay or prevent cancer, CVD, as well as complications of radiotherapy. Early pilot trials of senolytic drugs suggest they decrease SASP cells and inflammation in humans. Clinical trials of these drugs as single regimen or in combination are beginning or underway (127, 128). However, hitting a single target in SCAPs may increase off-targeting risk. For example, Navitoclax, the well-known BCL-2 inhibitor that hits a single or few SCAP nodes, causes substantial off-targeting effects on non-senescent cells and thereby making that drug “panolytic” (126, 129–131). To reduce off-targeting effects and increase the specificity of senolytic drugs, efforts are made to target more than one SCAP nodes, as evident by the using of combination therapy in clinical trials (NCT028749819, NCT02652052, NCT0463124, NCT02848131, NCT04210986, and NCT03675724). The combination therapy of Dasatinib + Quercetin, and Fisetin showed inhibitory effect on senescent cells *in vivo* and not on non-senescent cells (132, 133). The senolytic efficacy of the combination therapy Dasatinib + Quercetin was further validated in cultured human senescent cells isolated from the abdominal subcutaneous adipose tissue fragments from diabetic and obese patients after surgery. Treatment of the cells with Dasatinib + Quercetin in culture reduced the senescent cells by 70% within 2 days (134–138).

**Table 1:** FDA approved anti-cancer drugs inducing therapy-induced senescence.

**Type of drugs**	**Name**	**Senescence marker**	**References**
Topoisomerase poisons/inhibitors	Doxorubicin (Adriamycin)	p53, SA-β-gal, p21Cip1, p16INK4, Morphology, growth arrest, SASP (IL-8, VEGF)	(96–98)
	Daunorubicin	SA-β-gal, growth arrest	(99)
	Etoposide	SA-β-gal, p53, p21Cip1, growth arrest, p16INK4, SASP (IL-6, IL-8, IL-1β)	(100–102)
	Mitoxantrone	SASP, Growth arrest, SA-β-gal, yH2AX, morphology	(24, 103)
Alkylating agents	Busulfan	Growth arrest, SA-β-gal, p16INK4, p19INK4	(104–107)
mTOR inhibitors	Rapamycin (Sirolimus)	SA-β-gal, morphology	(108)
PARP inhibitor	Olaparib	Growth arrest, γH2AX, 53BP1, SA-β-gal, p21Cip1, p27Kip1, p15INK4, p16 INK4, p57, SASP (IL8)	(109, 110)
	Niraparib	Growth arrest, morphology, SA-β-gal, γH2AX	(110)
	Rucaparib	SA-β-gal	(111)
Proteasome inhibitors	Bortezomib	SA-β-gal, morphology	(112)
Monoclonal antibodies	Rituximab	Morphology, SA-β-gal	(113)
	Obinutuzumab	SA-β-gal	(114)
	Pertuzumab	SA-β-gal	(115)
	Trastuzumab	SA-β-gal, p15INK4, p16INK4	(115)
	Bevacizumab	SA-β-gal, p15INK4, p16INK4	(116)
	Ranibizumab	SA-β-gal, cathepsin D, amyloid β	(117)

## SASP Instigates CVD

CVD remain the most common age-related diseases worldwide and the leading cause of death in the aged individuals (139, 140). Studies of human samples and mouse models reveal that senescent cardiovascular cells accumulate at the site of the disease cardio-vasculature and leading to atherosclerosis, heart failure, arterial stiffness, and hypertension (43, 141). The persistent senescence of cardiovascular cells leads to CVD, however cardiovascular cell senescence is also required for the maintenance of cardiovascular homeostasis during embryonic development and wound healing (142). Importantly, with nutritional and growth factor deficiency, cardiovascular cells enter reversible quiescence (143). In the normal aging process, senescent cells accumulate in the cardiovascular system and predispose it to aging-related CVD (43). Following the onset of CVD, the microenvironment of the diseased tissues creates more cellular stress, and a second wave of disease-associated senescent cells is produced enhancing the disease process. Cells of the cardiovascular system including cardiomyocytes, endothelial, vascular smooth muscle, and immune cells (144), significantly contribute to the development and progression of CVD.

Cardiac metabolism has an important role in maintaining the heart's function and cardiovascular homeostasis (145). Cardiac aging is associated with a decreased angiogenic capacity (146), an increased fibrosis (147), metabolic maladaptation (148), cardiomyocyte senescence and dysfunction (149), all of which lead to cardiac remodeling and failure (150). Senescent cardiomyocytes exhibit the hallmarks of DNA damage, mitochondria dysfunction, contractile dysfunction, endoplasmic reticulum stress, hypertrophic growth, and SASP. In the heart, the senescence of cardiomyocytes is also regulated by non-cardiomyocyte cells (endothelial cells, fibroblasts, and immune cells). The senescence of cardiomyocytes also leads to phenotypic and functional changes in those non-cardiomyocyte cells and thereby contributing to cardiac aging and pathological remodeling (43, 151, 152). Nevertheless, the molecular mechanisms by which the senescence of cardiomyocytes is induced and regulated, as well as their interaction with the senescence of non-cardiomyocytes remain to be fully studied. Furthermore, how the local microenvironment of the heart, and how chromatin structure remodeling and DDR activated by cardiomyocytes contribute to the senescence of cardiomyocytes are not well-known. More studies are needed to determine the physiological and pathological functions of senescent cardiomyocyte during cardiac development, regeneration, and pathological remodeling, also to understand whether cardiomyocyte senescence has a role in cardiac aging and the related heart failure with preserved ejection fraction.

Endothelial cells form the inner layer of all blood vessels and communicate with the neighboring cells for tissue regeneration, and control low density lipoprotein (LDL) transcytosis and atherogenesis (153). Endothelial cell dysfunction is associated with the development of atherosclerotic plaques, and is tightly linked to endothelial senescence (154). In the atherosclerotic plaques from human coronary arteries, senescent endothelial cells with high beta-galactosidase activity, a marker of senescent cells, were detected (154). In the initial stages of atherogenesis, an increase of ox-LDL retention was observed in the subendothelial spaces. Senescent endothelial cells undergoing SASP express adhesion molecules including VCAM1 and ICAM1 and secrete various cytokines, leading to the recruitment of circulating monocytes, driving monocyte invasion. Consequently, circulating monocytes invade to the subendothelial spaces, take up the ox-LDL and are converted to foam cell macrophages (155). Both foam cells and SASP endothelial cells secrete a plethora of chemoattractant proteins including IL-1α, TNF -α, and MCP-1 which promote more immune cell recruitment and form atherosclerotic plaques. SASP endothelial cells also cause thrombus formation through the activation of PAI-1, a known marker of senescence (156).

Vascular smooth muscle cells also play a significant role in cardiovascular homeostasis. Vascular smooth muscle cell senescence and pro-inflammatory phenotype has been implicated in the development of CVD, progression of atherosclerosis, and an instigator of ischemic heart disease (157, 158). Compared to normal vascular smooth muscle cells, those isolated from human atherosclerosis exhibited a lower level of proliferation (159) and higher expression level of p16INK4, p21Cip1, hypo phosphorylated Rb, and SA-β-gal activity, suggesting the cells are undergoing SASP (160). Vazquez-Padron et al. reported that vascular smooth muscle cells derived from aged thoracic aortas have higher levels of platelet-derived growth factor receptor-alpha and acquire resistant to apoptosis induced by serum starvation or NO (161). Human vascular smooth muscle cells undergoing SASP had an inactivation of Sirt1, were increased in atherosclerosis (162), and vulnerable atherosclerotic plaque (163).

Patients over the age of 60 who have shorter leukocyte telomere length, a cellular senescence marker, showed 3.18-fold higher in mortality rate from heart failure (164). It is becoming clear that immunosenescence contributes to both innate and adaptive immune systems. Senescent T cells can produce many pro-inflammatory cytokines and chemokines and therefore they have pathogenic potential in CVD such as hypertension, atherosclerosis, myocardial infarction, and heart failure (165). In individuals with human immunodeficiency virus infection treated with a combination of antiretroviral therapy, we found that four components of SASP, including (1) telomere shortening-induced DNA damage and the subsequent induction of p53, p16INK4, and p21Cip1; (2) mitochondrial ROS induction; (3) inflammation; and (4) impairment of efferocytosis, were regulated by p90RSK-mediated ERK5 S496 phosphorylation in myeloid cells. We also found a key role of p90RSK-mediated ERK5 S496 phosphorylation in SASP-mediated atherosclerotic plaque formation (166, 167). Since cancer therapy can induce SASP (22, 54, 168), p90RSK-mediated ERK5 S496 phosphorylation may play a role in SASP induced by cancer therapy.

## Cardiovascular Risk Factors = Cancer Risk Factors, Because SASP Is Shared?

**Figure 3:**
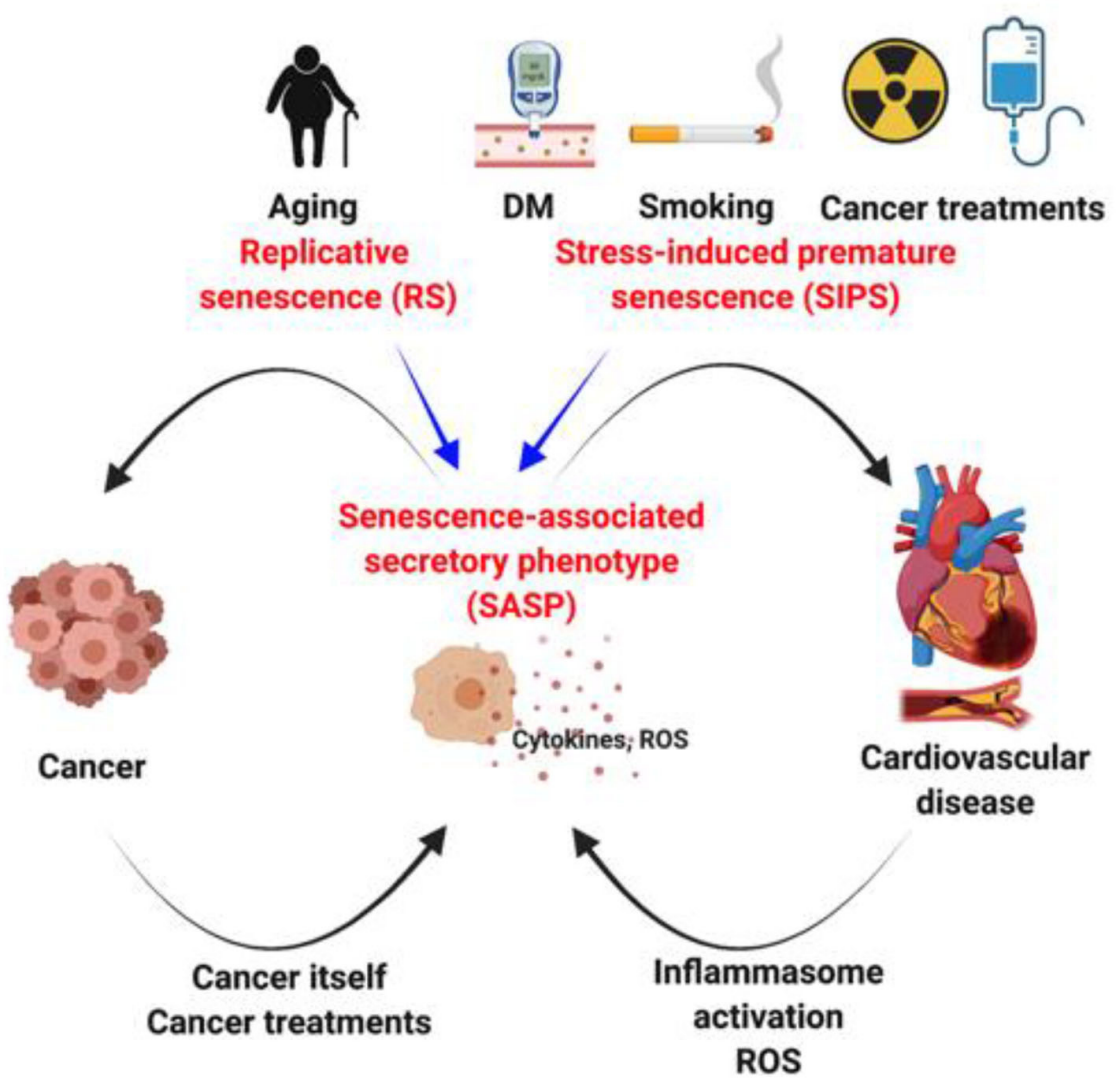
SASP as a hinge between cancer and cardiovascular disease. DM, Diabetes mellitus.

The clear epidemiological connection between aging, diabetes mellitus, smoking, cancer, and heart failure raised an obvious question about the pathological link among them (169). Ma et al. showed that shorter telomere length in diabetes mellitus patients is probably due to higher ROS production (170). Cigarette smoke extract increases ROS production and subsequently enhances p16 expression in the progenitor endothelial cells, leading to endothelial dysfunction (56, 171). The crucial role of p16 in smoking induced SASP and subsequent lung injury has also been suggested (172). These data suggest that ROS-mediated SASP induction can be a convergent point in the development of cancer and CVDs. As stated above, ROS is pivotal to initiate telomere DNA damage and the subsequent SASP induction. However, because telomere DNA damage-induced SASP is irreversible once established (17), antioxidant therapy may be no longer effective to attenuate SASP during the progression of cancer and CVD. As such, understanding the regulation of SASP is the key to understand not only the interconnections between cancer and CVD, but also age-related diseases such as diabetes mellitus, Alzheimer's disease, cataract, and chronic obstructive pulmonary disease (Figure 3).

## Conclusion

The crucial role of senescence in both cancer and CVD is becoming evident. However, the contribution of senescence to the interconnection between cancer and CVD remains unclear. In this review, we discuss the possible involvement of SASP, which can be instigated by various stresses, including cancer therapy, ROS, and pro-inflammatory cytokines, in the establishment of interconnection between cancer and CVDs. Especially, the new concept of senescence-associated stemness, a unique form of SASP, which may have a significant impact on determining the interplay between cancer and CVD, under highly stressful conditions such as cancer therapy, myocardial infarction, and heart failure. Therefore, although the involvement of senescence in cancer and CVD is a kind of old concept, the perspective of senescence is radically changing.

## Author Contributions

PB, NTL, and JA wrote a manuscript. ED edited and made critical suggestions for a manuscript. SK, LR, RA, JC, KS, AD, JH, and SL critically read the manuscript and provided suggestions for improving the manuscript contents. All authors contributed to the article and approved the submitted version.

## Funding

The research activity related to this review was partially supported by grants from the National Institutes of Health (NIH) to JA (AI-156921).

## Conflict of Interest

SL is an Advisory Board member of AstraZeneca, Beyond Spring Pharmaceuticals, STCube Pharmaceuticals.The remaining authors declare that the research was conducted in the absence of any commercial or financial relationships that could be construed as a potential conflict of interest.

## Publisher's Note

All claims expressed in this article are solely those of the authors and do not necessarily represent those of their affiliated organizations, or those of the publisher, the editors and the reviewers. Any product that may be evaluated in this article, or claim that may be made by its manufacturer, is not guaranteed or endorsed by the publisher.
